# Incidence of new-onset in-hospital and persistent diabetes in COVID-19 patients: comparison with influenza

**DOI:** 10.1016/j.ebiom.2023.104487

**Published:** 2023-02-28

**Authors:** Justin Y. Lu, Jack Wilson, Wei Hou, Roman Fleysher, Betsy C. Herold, Kevan C. Herold, Tim Q. Duong

**Affiliations:** a Department of Radiology, Albert Einstein College of Medicine and Montefiore Medical Center, Bronx, New York, United States; b Department of Family and Preventive Medicine, Stony Brook University, Stony Brook, New York, United States; c Department of Pediatrics and Microbiology-Immunology, Albert Einstein College of Medicine, Bronx, New York, United States; d Department of Immunobiology and Medicine, Yale University, New Haven, CT, United States

**Keywords:** Hyperglycemia, COVID-19, Inflammation, Cytokine storm, Predictive model

## Abstract

**Background:**

This study investigated the incidences and risk factors associated with new-onset persistent type-2 diabetes during COVID-19 hospitalization and at 3-months follow-up compared to influenza.

**Methods:**

This retrospective study consisted of 8216 hospitalized, 2998 non-hospitalized COVID-19 patients, and 2988 hospitalized influenza patients without history of pre-diabetes or diabetes in the Montefiore Health System in Bronx, New York. The primary outcomes were incidences of new-onset in-hospital type-2 diabetes mellitus (I-DM) and persistent diabetes mellitus (P-DM) at 3 months (average) follow-up. Predictive models used 80%/20% of data for training/testing with five-fold cross-validation.

**Findings:**

I-DM was diagnosed in 22.6% of patients with COVID-19 compared to only 3.3% of patients with influenza (95% CI of difference [0.18, 0.20]). COVID-19 patients with I-DM compared to those without I-DM were older, more likely male, more likely to be treated with steroids and had more comorbidities. P-DM was diagnosed in 16.7% of hospitalized COVID-19 patients versus 12% of hospitalized influenza patients (95% CI of difference [0.03,0.065]) but only 7.3% of non-hospitalized COVID-19 patients (95% CI of difference [0.078,0.11]). The rates of P-DM significantly decreased from 23.9% to 4.0% over the studied period. Logistic regression identified similar risk factors predictive of P-DM for COVID-19 and influenza. The adjusted odds ratio (0.90 [95% CI 0.64,1.28]) for developing P-DM was not significantly different between the two viruses.

**Interpretation:**

The incidence of new-onset type-2 diabetes was higher in patients with COVID-19 than influenza. Increased risk of diabetes associated with COVID-19 is mediated through disease severity, which plays a dominant role in the development of this post-acute infection sequela.

**Funding:**

None.

**Research in context**

**Evidence before this study**

We searched PubMed and medRxiv with the search terms “new-onset diabetes”, “post-COVID-19 sequelae”, “persistent diabetes”, “hyperglycemia”, “diabetes mellitus”, “SARS-CoV-2” and “influenza” for articles published between Dec 8, 2020 and Jul 7, 2022. Patients infected with SARS-CoV-2 are at higher risk of developing new persistent diabetes. Data on the new persistent diabetes associated with SARS-CoV-2 infection compared to a similar respiratory virus (influenza) and identification of risk factors for persistent diabetes in COVID-19 patients may draw clinical attention for the need for careful follow-up.

**Added value of this study**

New-onset type-2 diabetes at follow-up is seen in 16.7% of patients who are hospitalized for COVID-19 and who did not have a prior history of pre-diabetes and diabetes. The rates of diabetes are higher in hospitalized compared to non-hospitalized patients with COVID-19. Older patients who are male and with underlying major comorbidities are more likely to have new-onset diabetes persist after hospitalization. After adjusting for demographic factors and severity of illness, the incidence of post-infectious diabetes is similar between COVID-19 and influenza patients. The severity of illness rather than the respiratory virus per se is likely responsible for persistent diabetes and the increased incidence of newly diagnosed persistent diabetes likely reflects the incidence of severe COVID-19 observed particularly during the first wave of the pandemic.

**Implications of all the available evidence**

New-onset diabetes at follow-up is seen in 16.7% of patients who are hospitalized with COVID-19 and who did not have a prior history of pre-diabetes and diabetes. The incidence of new-onset diabetes during hospitalization was 3.96 times (adjusted odds ratio) higher in patients with COVID-19 compared to those with influenza but only 1.24 (adjusted odds ratio) times higher at follow-up. Our findings suggest that the portion of increased risk of diabetes associated with COVID-19 is mediated through disease severity, which plays a dominant role in the development of this post-acute infection sequela. Identification of risk factors for P-DM could enable the need for careful follow-up in COVID-19 patients.

## Introduction

The clinical course of SARS-CoV-2 infection is well-documented to be more severe in patients with pre-existing diabetes.^1^, ^2^, ^3^, ^4^, ^5^, ^6^, ^7^ Metabolic decompensation occurs frequently in COVID-19 patients with diabetes and tightening metabolic control improves outcomes.^8^ Conversely, SARS-CoV-2 infection has also been proposed to trigger new-onset diabetes.^3^, ^4^, ^5^^,^^9^ Several reports have drawn attention to new-onset diabetes among patients hospitalized with COVID-19,^10^, ^11^, ^12^, ^13^ which some have speculated may be the direct result of viral infection of insulin producing β cells, although SARS-CoV-2 viral particles have not been identified in β cells.^14^ The more common observation of diabetes in older hospitalized patients with COVID-19 suggests that inflammatory responses to infection together with obesity leads to insulin resistance and metabolic decompensation. In addition, COVID-19 treatments (e.g., glucocorticoids), may unmask latent diabetes because of their metabolic effects.

It is unclear whether new-onset diabetes diagnosed during COVID-19 persists after resolution of the acute infection. The effects of inflammatory mediators on β-cell dysfunction or insulin resistance should resolve with clinical improvement. Alternatively, there may be metabolic memory and thus the effects may persist even after the acute infection resolution. It is also not known whether new-onset diabetes that persists or presents following recovery from the acute viral illness is a consequence of SARS-CoV-2 virus, patient clinical profile, and/or hospital course and whether the incidence of this post-infectious sequela differs from what occurs following other severe respiratory viral infections such as influenza. The course of new-onset diabetes in COVID-19 patients may suggest pathologic mechanisms that contribute to its appearance.

The goals of this study were to determine whether COVID-19 related new-onset type-2 diabetes occurred more frequently among patients with COVID-19 compared to influenza, whether it was transient or persistent among those diagnosed in-hospital, and whether it also presented as a post-COVID-19 sequela. Outcomes were adjusted with covariates (age, sex, and major comorbidities) using odds ratios as well as compared with propensity matched controls. We also analyzed the incidence of diabetes across the pandemic and during the peak of each COVID-19 wave, and between hospitalized and non-hospitalized COVID-19 patients. Predictive models were used to identify and compare risk factors associated with post-COVID-19 or post-influenza new-onset diabetes.

## Methods

### Ethics

This study was approved by the Einstein-Montefiore Institutional Review Board (#2021-13658) with an exemption for informed consent.

### Data sources

Health data came from the Montefiore Health System with 15 hospitals and medical centers located in New York Metropolitan area in the Bronx and the lower Westchester County (∼10 miles diameter), which serves a large diverse patient population including many patients with lower social economic status. Electronic medical records were extracted automatically as described previously.^15^, ^16^, ^17^, ^18^, ^19^, ^20^ De-identified health data were obtained for research after standardization to the Observational Medical Outcomes Partnership (OMOP) Common Data Model (CDM) version 6. OMOP CDM represents healthcare data from diverse sources, which are stored in standard vocabulary concepts,^21^ allowing for the systematic analysis of disparate observational databases, including data from the electronic medical record (EMR), administrative claims, and disease classifications systems (e.g., ICD-10, SNOWMED, LOINC, etc.). ATLAS, a web-based tool developed by the Observational Health Data Sciences and Informatics (OHDSI) community that enables navigation of patient-level, observational data in the CDM format, was used to search vocabulary concepts and facilitate cohort building. Data were subsequently exported and queried as SQLite database files using the DB Browser for SQLite (version 3.12.0). For the variables extracted, chart reviews of a subset (N > 100) of data were performed to verify data accuracy and completeness.

### Participants

From March 11, 2020 to Feb 20, 2022, there were 35,644 COVID-19 positive patients, identified by polymerase-chain-reaction (PCR) test. From Jan 2018 to Feb 20, 2022, there were 12,354 hospitalized patients who tested positive for influenza without a positive COVID-19 PCR test. Using pre-COVID-19 pandemic data, we excluded patients with type-2 diabetes or prediabetes ICD10 diagnosis codes, on diabetes medications regardless of diabetes or prediabetes diagnosis, with A1c of 5.7–6.5% (pre-DM) or ≥6.5% (DM) prior to admission, two fasting glucoses of 100–125 mg/dl (pre-DM), a random glucose of 140–199 mg/dl (pre-DM), two fasting glucose readings ≥126 (DM) or two random glucoses of ≥200 mg/dl prior to admission (DM).

### Variables

Demographic data included age, sex, race, and ethnicity were collected via EMR. Preexisting comorbidities included body mass index (BMI), congestive heart failure (CHF), chronic kidney disease (CKD), hypertension, chronic obstructive pulmonary disease (COPD) and asthma that were designated by ICD10 codes at admission or prior. Steroid treatment, hospitalization status, intensive-care-unit (ICU) admission, and mortality were also extracted. Admission vital signs and laboratory data collected from hospitalized patients included temperature, systolic blood pressure (SBP), oxygen saturation (SPO_2_), lactate dehydrogenase (LDH), brain natriuretic peptide (BNP), creatinine (Cr), C-reactive protein (CRP), ferritin (FERR), D-dimer (DDIM), troponin-T (TNT), alanine aminotransferase (ALT), white-blood-cell count (WBC), lymphocyte count (Lymph), and prothrombin time (PT).

Overall, 64% of patients in this study returned to the health system ∼3 months after diagnosis (mean = 83 and 87 days for COVID-19 and influenza patients, respectively). Data was collected at admission and the follow-up visit.

### Analysis of COVID-19 waves/strains

Predominant SARS-CoV-2 waves were estimated based on New York State Department of Health data https://coronavirus.health.ny.gov/covid-19-variant-data (assessed Nov 22, 2022). Waves were defined by daily test positivity 5% above baseline that lasted at least 10 days in Bronx, New York.^22^ By this definition, the first wave spanned from March 8, 2020, to May 25, 2020, the alpha wave from December 6, 2020 to April 5, 2021, the Delta wave from July 6, 2021 to December 14, 2021, and the Omicron wave from December 15, 2021 to January 24, 2022.

### Outcomes

The primary outcomes analyzed were the incidence of new-onset type-2 diabetes mellitus while in the hospital (I-DM), and, for patients who returned to the hospital system, new-onset persistent diabetes mellitus (P-DM) at ∼3-month follow-up using above-defined criteria, grouped by SARS-CoV-2 or influenza infection. Outcomes by months and by waves across the pandemic were also analyzed.

#### Predictive model/sample size

Logistic regression was used to build the predictive model. Sample size was based on availability of subjects. Univariable analysis was performed using each variable separately (demographics, comorbidities, and lab values, except FERR, BNP and A1c which had data missing from >15% of patients). All data were used except those with missing data >15%. Imputation was done for data missing <15%. The top 8 laboratory variables out of 14 (all extracted laboratory variables in Table 1) were first identified based on P-values. These top 8 laboratory variables out of 14 were then combined with all demographics, comorbidities collected to the logistic model to predict P-DM. This approach was adopted to avoid overfitting. Model performance was evaluated using area under the receiver operating characteristic curve with five-fold cross validation.^17^^,^^23^

**Table 1:** COVID-19 patient characteristics of (A) patients during hospitalization (B) hospitalized patients at follow-up, (C) hospitalized patients with in-hospital new-onset diabetes at follow-up, and (D) non-hospitalized patients at follow-up.

COVID-19	(A) Hospitalized patients during hospitalization (N = 8216)		(B) All hospitalized patients at follow-up (N = 4982)		(C) Subset of hospitalized patients with in-hospital DM at follow-up (N = 1034)		(D) Non-hospitalized patients at follow-up (N = 1942)	
	New-onset I-DM (N = 1854, 22.6%)	No I-DM (N = 6362, 77.4%)	Post-COVID P-DM (N = 834, 16.7%)	No P-DM (N = 4148, 83.3%)	Post-COVID P-DM (N = 383, 37.0%)	No P-DM (N = 651, 63.0%%)	Post-COVID P-DM (N = 142, 7.3%)	No P-DM (N = 1800 92.7%)
Age, yo, median (IQR)	66 (55, 78)	41 (30, 59)	62 (50, 75)	45 (32, 62)	66 (55, 77)	63 (53, 76)	54.9 ± 18.3	40.9 ± 16.0
Female, n (%)	818 (44.1%)	3896 (61.2%)	405 (48.6%)	2519 (60.7%)	161 (42.0%)	311 (47.8%)	74 (52.1%)	1250 (69.4%)
White, not hispanic	191 (10.3%)	610 (9.6%)	92 (11.0%)	373 (9.0%)	50 (13.1%)	70 (10.8%)	18 (12.7%)	245 (13.6%)
Black, not hispanic	572 (30.9%)	1930 (30.3%)	280 (33.6%)	1266 (30.5%)	129 (33.7%)	227 (34.9%)	43 (30.3%)	475 (26.4%)
Hispanic	791 (42.7%)	2957 (46.5%)	354 (42.4%)	1941 (46.8%)	171 (44.6%)	272 (41.8%)	48 (33.8%)	594 (33.0%)
Other	300 (16.2%)	865 (13.6%)	108 (12.9%)	568 (13.7%)	33 (8.6%)	82 (12.6%)	33 (23.2%)	486 (27.0%)
BMI	29.0 (25.1, 34.3)	28.0 (23.9, 32.8)	28.9 (24.8, 34.4)∗∗	28.7 (24.6, 33.2)	29.1 (25.1, 34.9)	29.8 (26.1, 34.4)	29.5 (26.6, 33.9)	28.2 (24.6, 32.1)
**Comorbidities, n (%)**								
CHF	221 (11.9%)∗∗∗	384 (6.0%)	167 (20.0%)∗∗∗	279 (6.7%)	88 (23.0%)∗∗∗	72 (11.1%)	15 (10.6%)∗∗∗	49 (2.7%)
CKD	106 (5.7%)∗∗	283 (4.4%)	81 (9.7%)∗∗∗	204 (4.9%)	34 (8.9%)∗	32 (4.9%)	10 (7.0%)∗∗∗	43 (2.4%)
Hypertension	487 (26.3%)∗∗∗	1436 (22.6%)	352 (42.2%)∗∗∗	1068 (25.7%)	154 (40.2%)∗∗	196 (30.1%)	55 (38.7%)∗∗∗	295 (16.4%)
COPD/Asthma	122 (6.6%)∗∗∗	1088 (17.1%)	109 (13.1%)∗∗	726 (17.5%)	31 (8.1%)	61 (9.4%)	19 (13.4%)	245 (13.6%)
**Steroid usage**	462 (24.9%)∗∗∗	423 (6.6%)	125 (15.0%)∗∗∗	430 (10.4%)	87 (22.7%)	187 (28.7%)		
**ICU admission**	77 (4.2%)	270 (4.2%)	21 (2.5%)∗∗∗	222 (5.4%)	17 (4.4%)	43 (6.6%)		
**In-hospital death**, **n (%)**	313 (16.9%)∗∗∗	167 (2.6%)	na	na	na	na		
**Lab at admission**, median (IQR)								
CRP	8.3 (3.6, 16.0)∗∗∗	3.5 (0.9, 8.9)	6.1 (2.2, 13.7)∗∗∗	4.6 (1.2, 10.5)	6.8 (2.6, 5.1)	7.7 (3.08, 15.2)		
Ferritin	647 (308, 1305)∗∗	355 (131, 921)	559 (235, 1302)∗	441 (168, 1053)	631 (292, 1292)	615 (276, 1235)		
LDH	370 (276, 530)∗∗∗	283 (213, 393)	330 (251, 477)∗∗	304 (227, 429)	351 (260, 508)	360 (267, 516)		
BNP	60 (15, 243)	60 (14, 219)	86 (27, 647)∗∗∗	60 (13, 171)	66 (22, 410)	60 (14, 187)		
Cr	1.06 (0.81, 1.50)∗∗∗	0.83 (0.70, 1.06)	1.00 (0.80, 1.36)∗∗∗	0.84 (0.70, 1.11)	1.06 (0.81, 1.52)	1.02 (0.79, 1.37)		
D-dimer	1.33 (0.74, 2.80)∗	0.94 (0.50, 1.98)	1.22 (0.66, 2.87)∗∗∗	1.00 (0.55, 2.05)	1.31 (0.72, 3.19)	1.29 (0.75, 2.46)		
TNT	0.01 (0.01, 0.03)	0.01 (0.01, 0.01)	0.01 (0.01, 0.02)	0.01 (0.01, 0.01)	0.01 (0.01, 0.03)	0.01 (0.01, 0.02)		
ALT	31 (20, 51)∗∗∗	23 (15, 37)	26 (17, 43)	25 (16, 41)	28 (18, 47)	31 (20, 51)		
LYMPH	1.1 (0.7, 1.4)∗∗∗	1.2 (0.8, 1.8)	1.1 (0.8, 1.5)	1.2 (0.8, 1.7)	1.1 (0.8, 1.5)	1.1 (0.7, 1.4)		
WBC	7.2 (5.4, 10.0)∗∗∗	6.4 (4.8, 8.6)	6.8 (5.0, 9.5)∗∗∗	5.7 (5.4, 6.2)	7.5 (5.6, 10.5)∗	6.9 (5.2, 9.6)		
PT	13.8 (13.1, 14.9)∗∗	13.6 (13.0, 14.4)	13.9 (13.3, 15.1)∗∗∗	13.6 (13.1, 14.4)	14.0 (13.3, 15.1)	13.8 (13.1, 14.7)∗		
SBP	131 (116, 147)	129 (117, 144)	134 (120, 150)∗∗∗	129 (116, 144)	133 (119, 150)	131 (116, 147)∗		
SPO2	95 (91, 98)∗∗∗	98 (96, 99)	97 (94, 98)∗∗∗	98 (96, 99)	95 (92, 98)	95 (92, 98)		
TEMP	98.7 (98.1, 99.7)∗∗∗	98.6 (98.2, 99.4)	98.7 (98.1, 99.6)∗	98.7 (98.2, 99.5)	98.7 (98.1, 99.7)	98.7 (98.2, 99.7)		

Laboratory test data were obtained at admission. SD: standard deviation. ∗p < 0.05, ∗∗p < 0.01, ∗∗∗p < 0.001 between new diabetes and no diabetes within each group.

To further investigate potential causal effects of virus type on outcomes (P-DM), we performed inverse probability weighting-based mediation analysis using the “MEDIATION” package in R software. CRP, LDH and DDIM were evaluated as the mediators in three separate models. ORs with 95% confidence intervals based on 1000 nonparametric Bootstrap simulations were calculated. Average direct effects of virus type and mediated proportions were calculated.

#### Exploratory analysis

Interrupted time series analysis was performed to investigate whether COVID-19 vaccine rollout affected I-DM and P-DM incidence.^24^ Sensitivity analysis of model performance metrics was also performed for different levels of prevalence of P-DM.

#### Propensity score matching

Given the age differences between some groups, the adjustment with age as a covariate might not be adequate. Thus, ORs were calculated using propensity score matching on age and sex. We used 5:1 nearest neighbor propensity score matching without replacement with a propensity score estimated using logistic regression of the virus type on age and sex (R package “matchit”). After matching, 306 flu patients were successfully matched to 1530 COVID patients. Standardized mean differences for age and sex were 0.11 and 0.02 respectively indicating adequate balance.

### Statistical analysis methods

Statistical analysis was performed using Python, R and SAS (Version 9.4, Cary, NC, USA). Group comparison for categorical variables used χ^2^ or Fisher's exact tests, and for continuous variables used Mann–Whitney U test. P values less than 0.05 were considered statistically significant. Odds ratio was calculated with age, sex, ICU status, and admission CRP, LDH and D-Dimer as surrogates for severity as covariates using logistic regression. Comparisons of variables across different waves used ANOVA. P values for laboratory values were not adjusted for multiple comparisons due to the exploratory nature of this study.

### Role of funder

Not applicable.

## Results

### Rates of I-DM and P-DM are higher following COVID-19 than influenza viral infection

**Fig. 1:**
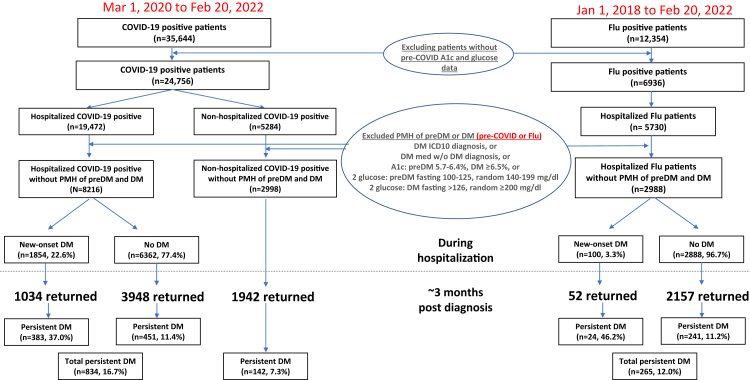
Patient selection flowchart. PMH: past medical history, DM: diabetes. Hospitalized influenza patient demographics pre (2018 and 2019) and post (2020 to Feb 2021) pandemic were similar and were thus combined. Note that there were more hospitalized (n = 8216) than non-hospitalized COVID-19 patients (n = 2998) in our cohort because the exclusion/inclusion criteria used to confirm no history of preDM and DM and many non-hospitalized patients did not have these detailed data and thus excluded.

Fig. 1 shows the patient selection flowchart. Among 19,472 patients hospitalized with COVID-19 who met the inclusion criteria, 8216 were identified as not having a history of pre-diabetes or diabetes using the predefined criteria. Among these, 1854 (22.6%) were diagnosed with type-2 diabetes during their hospitalization (I-DM). 1034 of those with a diagnosis of I-DM returned for follow-up and 383 (37.0%) still had diabetes (P-DM). Notably, among the hospitalized patients with COVID-19 who did not have diabetes while in the hospital, 3948 returned and an additional 451 (11.4%) were diagnosed with P-DM. Combining patients with and without I-DM, P-DM at follow-up was documented in 16.7% (n = 834). There were 2998 non-hospitalized COVID-19 patients without history of prediabetes/diabetes and 1942 returned; only 142 (7.3%) had P-DM at follow-up, which was significantly less than the rate among hospitalized patients with COVID-19 (7.3% vs 16.7, 95% CI of difference [0.078, 0.11], p < 0.001).

We identified 2988 hospitalized influenza patients who met the inclusion criteria. Only 100 (3.3%) had I-DM. Thus, hospitalized patients with COVID-19 were much 6.8 times more likely to develop I-DM compared to the influenza counterparts (22.6% vs 3.3%, p < 0.001, with OR = 3.96, [95% CI:3.2, 4.96], p < 0.001, adjusted for age, sex, comorbidities). However, 52 of the 100 who had influenza returned for follow-up and diabetes persisted in 24 (46.2%), which was higher than observed with COVID-19 (37% vs 46.2%, p = 0.0035). Among the hospitalized influenza patients who did not have I-DM, 2157 returned and 241 (11.2%) had P-DM, which is comparable to the rate of P-DM among COVID-19 patients without I-DM (11.4%). When those with or without I-DM are combined, hospitalized patients with COVID-19 were more likely to develop P-DM compared to the influenza counterparts (1.39 times from 16.7% vs 12.0%, p < 0.0001, with adjusted OR = 1.24, [95% CI:1.07, 1.45], p < 0.001).

Overall, 64% of patients returned to the health system ∼3 months after diagnosis (mean = 83 and 87 days for COVID-19 and influenza patients, respectively). There were no significant differences in demographics, race, ethnicity, and major comorbidities (p > 0.05) between patients who did or did not return for follow up visits.

### Features of COVID-19 patients with and without I-DM

Table 1A compares the COVID-19 patient characteristics with and without I-DM during hospitalization. Patients with I-DM were older, less likely to be female, had a higher prevalence of CHF, CKD, and hypertension, a lower prevalence of COPD/asthma (all p < 0.001), but the BMI was not significantly different (p = 0.063) vs those without I-DM. More I-DM patients were treated with steroids (OR = 3.97 [95% CI: 3.33–4.74, p < 0.05]) after adjustment for age, sex and ICU admission. Mortality rate was higher among I-DM patients (p < 0.001).

Patients with I-DM had higher CRP, ferritin, LDH, Cr, D-dimer ALT, WBC, and PT (p < 0.05) and lower SPO_2_ and lymphocyte counts (p < 0.001) on admission compared to those without I-DM, findings consistent with greater disease severity.

### Features of COVID-19 patients with P-DM

Table 1B shows data for the hospitalized COVID-19 patients who returned for follow-up. Patients with P-DM were older, less likely to be female, and had higher prevalence of CHF, CKD, hypertension, and a lower prevalence of COPD/asthma. The BMI was higher in those with P-DM compared to those without. More P-DM patients had been treated with steroids, but the (OR =1.19 [95% CI: 0.93–1.52], p > 0.05) was not significantly different after adjustment for age, sex and ICU admission. Patients who were subsequently diagnosed with P-DM also had significantly higher admission laboratory data reflecting inflammation and disease severity (CRP, ferritin, LDH, BNP, Cr, D-dimer, WBC, PT, and SBP) and lower SPO_2_ compared to those without P-DM (all p < 0.05).

Table 1C compares the COVID-19 patients who returned for follow-up limited to the subgroup who were diagnosed with I-DM to identify factors that might predict those whose diabetes was persistent. Notably, I-DM was transient in most patients (63.0%). Those in whom diabetes persisted had higher prevalence of CHF, CKD, and hypertension. However, age, sex, steroid use, and most laboratory values (at admission) were not significantly different between those with transient versus persistent DM.

Similar to hospitalized patients, non-hospitalized COVID-19 patients with P-DM were older, less likely female, and had higher prevalence of CHF, CKD, and hypertension (all p < 0.001), but a similar prevalence of COPD/asthma and BMI compared to those without P-DM (Table 1D). Laboratory tests were generally not performed for non-hospitalized patients.

### Features of hospitalized influenza patients with and without I-DM and/or P-DM

Table 2A shows the influenza patient characteristics during hospitalization. Similar to the patients with COVID-19 and I-DM, those with influenza and I-DM were markedly older and had a higher prevalence of most major comorbidities compared to those without. More I-DM patients were treated with steroids (p < 0.001). ICU admission rates were low but not statistically different between groups. The I-DM cohort had a higher mortality rate. Laboratory data was not analyzed because the sample size of influenza patients with I-DM was small.

**Table 2:** Hospitalized influenza patient characteristics (A) during hospitalization and (B) at follow-up.

Influenza	(A) During hospitalization (N = 2988)		(B) at follow-up (N = 2209)	
	New-onset I-DM (N = 100, 3.3%)	No I-DM (N = 2888, 96.7%)	Post-influenza P-DM (N = 265, 12.0%)	No P-DM (N = 1944, 88.0%)
Age, yo, median (IQR)	62.5 (51, 72)	27 (12, 41)	50 (30, 63)	26 (11, 39)
Female, n (%)	48 (48.0%)	1178 (40.8%)	180 (67.9%)	1203 (61.9%)
White, not hispanic	9 (9.0%)	149 (5.2%)	19 (7.2%)	100 (5.1%)
Black, not hispanic	38 (38.0%)	770 (26.7%)	93 (35.1%)	518 (26.6%)
Hispanic	37 (37.0%)	1488 (51.5%)	128 (48.3%)	966 (49.7%)
Other	16 (16.0%)	481 (16.7%)	25 (9.4%)	360 (18.5%)
BMI	29.4 (24.1, 33.6)∗∗∗	25.6 (19.8, 30.9)	29.6 (25.1, 34.7)∗∗∗	25.0 (19.2, 30.4)
**Comorbidities, n (%)**				
CHF	17 (17.0%)∗∗∗	87 (3.0%)	34 (12.8%)∗∗∗	40 (2.1%)
CKD	8 (8.0%)∗∗	82 (2.8%)	22 (8.3%)∗∗∗	62 (3.2%)
Hypertension	31 (31.0%)∗∗∗	403 (14.0%)	118 (44.5%)∗∗∗	263 (13.5%)
COPD/Asthma	34 (34.0%)	865 (30.0%)	114 (43.0%)∗∗∗	630 (32.4%)
**Steroid**	32 (32.0%)∗∗∗	190 (6.6%)	42 (15.8%)∗∗∗	120 (6.2%)
**ICU admission**	0 (0%)	3 (0.1%)	1 (0.4%)	2 (0.1%)
**In-hospital death, n (%)**	6 (6.0%)∗∗∗	15 (0.5%)	na	na
**Lab at admission,** median (IQR)				
CRP			0.65 (0.525, 7.37)∗∗∗	1.55 (0.575, 5.7)
Ferritin			506 (113, 1043)∗∗∗	140 (41, 630)
LDH			178 (174, 183)∗∗∗	322 (239, 403)
BNP			196 (68.2, 525)	95 (60, 368)
Cr			0.81 (0.7, 1.1)∗	0.78 (0.6, 1)
D-dimer			0.535 (0.485, 0.575)∗∗∗	0.78 (0.665, 0.885)
TNT			0.01 (0.01, 0.01)	0.01 (0.01, 0.01)
ALT			17 (13, 28)∗∗	20 (13, 28)
LYMPH			0.9 (0.6, 1.5)	0.9 (0.6, 1.5)
PT			13.8 (13.2, 14.6)	13.9 (13.4, 14.5)
SBP			132 (115, 146)∗∗∗	117 (108, 129)
SPO2			97.5 (96, 99)∗∗∗	98 (97, 100)
TEMP			99 (98.3, 101)	99.2 (98.4, 101)
WBC			6.8 (4.8, 9.0)	6.5 (5.0, 8.5)

Positive influenza test (but no positive COVID-19 PCR tests) from Jan 1, 2018 to Feb 20, 2022. ∗p < 0.05, ∗∗p < 0.01, ∗∗∗p < 0.001 between new diabetes and no diabetes within each group. Laboratory test data were obtained at admission.

Table 2B shows the influenza patient characteristics at follow-up. Patients with P-DM were older and had higher prevalence of most major comorbidities. More P-DM patients were treated with steroids. Inflammatory markers including CRP, ferritin, LDH, D-Dimer were significantly higher at admission in the patients with influenza diagnosed with P-DM compared to those without P-DM.

The similarities of demographic and laboratory features with P-DM following COVID-19 (Table 1B) and influenza (Table 2B) suggested that differences in the rates might not be a feature of the virus itself but other clinical factors. Consistent with this notion we found that the OR of P-DM was not significantly different in hospitalized COVID-19 compared to influenza patients after adjusting for age and sex (OR = 1.14 [95% CI: 0.74–1.77], p = 0.61) without or with laboratory surrogates of disease severity (LDH, CRP and D-dimer) (OR = 0.90 [95% CI = 0.64,1.28], p = 0.56) (unmatched analysis). When analysis was performed using propensity score matching, the corresponding results were similar, namely, OR = 1.29 (95% CI: 0.93–1.76, p = 0.11) and 1.32 (95% CI = 0.94, 1.77, p = 0.13), respectively. P-DM ORs greater than unity, but with only trending p values toward significance suggest that small sample size and heterogeneity could contribute to non-significance.

### Prediction of diabetes with COVID-19 or influenza

#### Model development/specification

Logistic regression was used to build the predictive model. The top 8 laboratory variables were first identified based on P-values. These top 8 laboratory variables were then combined with demographics, comorbidities to the logistic model to predict P-DM.

#### Model performance

Predictive models were used to identify clinical variables associated with P-DM for COVID-19 or influenza patients who were hospitalized (Table 3). Using admission data, the significant top predictors were I-DM, age, CHF, steroid, ICU, and D-dimer for hospitalized COVID-19 patients, and age, I-DM, lymphocyte, BMI, and ALT for hospitalized influenza patients. Prediction of P-DM for the COVID-19 cohort yielded 71.42% AUC (95% CI [0.70,0.84]), 78.28 accuracy (95% CI [0.77,0.83]), 96.95 specificity (95% CI [0.96,0.99]), 14.05 sensitivity (95% CI [0.11,0.26]), and 51.66% positive predictive value (95% CI [0.58,0.91]). Prediction of P-DM for the influenza cohort yielded 66.70 AUC (95% CI [0.57,0.89]), 74.44 accuracy (95% CI [0.61,0.81]), 92.26% specificity (95% CI [0.83,0.97]), 22.14 sensitivity (95% CI [0.05,0.37]) and 48.0% positive predictive value (95% CI [0.14,0.79]). AUC curves for the test datasets are shown in Supplemental Fig. S1. Sensitivity analysis of the predictive models for different levels of prevalence of P-DM from 15% to 40% (Supplemental Table S1) showed that performance metrics were similar across different prevalence of P-DM.

**Table 3:** Prediction model performance indices and top predictors of P-DM for (A) hospitalized COVID-19 patients and (B) hospitalized influenza patients.

(A) Hosp. COVID-19 pts			(B) Hosp. influenza pts		
Variables	P Values	OR [95% CI]	Variables	P Values	OR [95% CI]
I-DM	**<0.00010**	3.32 [2.62,4.21]	Age	**0.00069**	1.02 [1.01,1.04]
Age	**<0.00010**	1.02 [1.01,1.02]	I-DM	**0.0028**	9.15 [0.3,272.71]
CHF	**<0.00010**	2 [1.49,2.67]	LYMPH	**0.0050**	1.44 [1.12,1.87]
Steroid	**0.018**	0.71 [0.53,0.94]	BMI	**0.0057**	1.07 [1.02,1.12]
ICU	**0.022**	0.51 [0.28,0.88]	ALT	**0.017**	0.98 [0.96,0.99]
D-dimer	**0.023**	1.03 [1.00,1.06]	CHF	0.054	2.13 [0.98,4.64]
BMI	0.081	1.01 [1.00,1.02]	COPD	0.076	1.7 [0.94,3.08]
Hypertension	0.083	1.24 [0.97,1.58]	SBP	0.13	1.01 [1,1.02]
PT	0.092	1.02 [1.00,1.04]	SPO2	0.13	0.94 [0.87,1.02]
Sex	0.14	0.85 [0.68,1.06]	ICU	0.14	1.35 [0.72,2.5]
CKD	0.16	1.33 [0.88,1.99]	WBC	0.16	0.94 [0.85,1.02]
TEMP	0.31	1.04 [0.96,1.13]	Steroid	0.21	0.63 [0.29,1.29]
LDH	0.40	0.99 [0.99,1.01]	CKD	0.28	0.61 [0.24,1.48]

Values in bold indicate p < 0.05. Note that prediction was not done for hospitalized influenza with I-DM because of small sample size.

For the mediation analysis, average direct effects of virus type were not significant with 95% confidence intervals, ranging from −0.03 to 0.06 for disease severity (CRP, LDH and DDIM as surrogates) as mediators (all p > 0.45), and all the mediated proportions were also not significant (all p > 0.4). Therefore, the mediation effects of CRP, LDH, and DDIM are not significant.

### Rates of diabetes rates across the COVID-19 pandemic

**Fig. 2:**
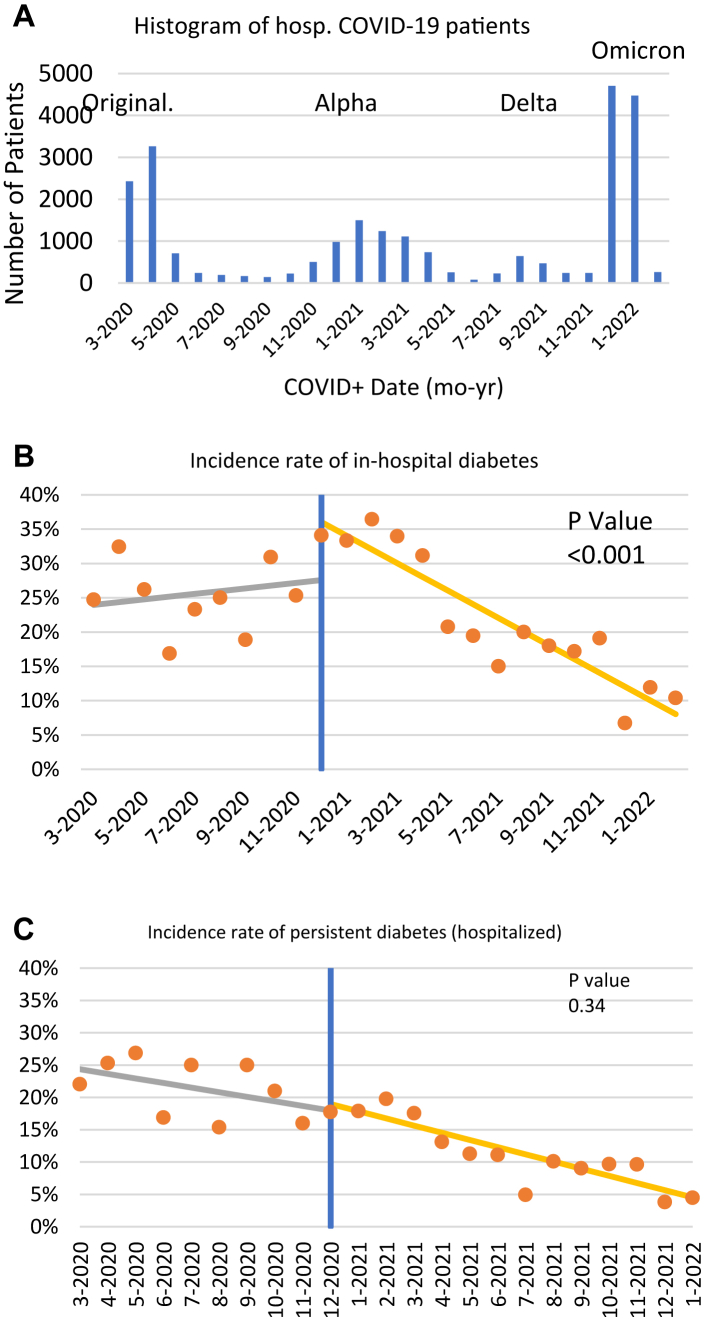
Incidences across the pandemic. (A) Number of hospitalized COVID-19 patients, interrupted time series analysis for (B) I-DM and (C) P-DM incidence. Blue lines indicate the date of the first vaccine rollout (Dec 14, 2021). P values indicate statistical significance value between incidence rate of diabetes before and after COVID-19 vaccine rollout.

To further distinguish the importance of disease severity or the virus itself leading to P-DM, we analyzed data over time during the pandemic. The prevalent strains varied in their clinical severity: original strain > Alpha > Delta > Omicron (Fig. 2A). To investigate whether COVID-19 vaccine rollout affected I-DM and P-DM incidence, interrupted time series analysis was performed. The I-DM incidence was high and time invariant till roughly when vaccine became available (Fig. 2B) and incidence of I-DM was significantly different before and after COVID-19 vaccine rolled out (p < 0.001. Incidence of P-DM decreased across the pandemic more steadily across waves (Fig. 2C), but incidence of P-DM was not significantly different before and after COVID-19 vaccine rolled out (p = 0.34).

There were general decreases in the incidence of I-DM and P-DM across the 4 waves (p < 0.001, Table 4). Age, sex, and comorbidities were not significantly different across waves. There were some differences in race and steroid use across waves. In the I-DM cohort, ICU admission was highest in the Omicron wave (p < 0.0001). Importantly, mortality rate decreased across waves (p < 0.0001).

**Table 4:** Incidence of I-DM and P-DM and their patient profiles grouped by different waves.

	I-DM					P-DM				
	Original	Alpha	Delta	Omicron	P value	Original	Alpha	Delta	Omicron	P value
Incidence of new DM	538/1863 (28.9%)	657/1903 (34.5%)	161/878 (18.3%)	198/2329 (8.5%)	<0.0001	356/1474 (24.2%)	260/1413 (18.4%)	49/547 (9.0%)	26/689 (3.8%)	<0.0001
Age, median (IQR) (yo)	54 (39, 68)	54 (36, 68)	39 (28, 55)	37 (27, 54)		57 (41, 71)	56 (41, 71)	40 (29, 58)	37 (26, 55)	
Female, n (%)	213 (39.6%)	312 (47.5%)	73 (45.3%)	89 (44.9%)		167 (46.9%)	136 (52.3%)	27 (55.1%)	10 (38.5%)	
White, not Hispanic	39 (7.2%)	76 (11.6%)	19 (11.8%)	27 (13.6%)		35 (9.8%)	42 (16.2%)	11 (22.4%)	3 (6.1%)	
Black, not Hispanic	201 (37.4%)	166 (25.3%)	49 (30.4%)	70 (35.4%)		146 (41.0%)	70 (26.9%)	18 (36.7%)	13 (26.5%)	
Hispanic	209 (38.8%)	281 (42.8%)	71 (44.1%)	82 (41.4%)		145 (40.7%)	117 (45.0%)	16 (32.7%)	7 (14.3%)	
Other	89 (16.5%)	134 (20.4%)	22 (13.7%)	19 (9.6%)		30 (8.4%)	31 (11.9%)	4 (8.2%)	3 (6.1%)	
BMI	29.1 (25.2,33.7)	28.7 (24.5,33.3)	28.9 (24.2,33.3)	28.1 (23.6,32.3)	0.74	28.9 (25.0, 33.6)	28.5 (24.4, 33.1)	28.8 (24.8, 33.5)	27.5 (23.5, 32.3)	0.22
**Comorbidities, n (%)**										
CHF	61 (11.3%)	84 (12.8%)	11 (6.8%)	25 (12.6%)	0.20	72 (20.2%)	56 (21.5%)	4 (12.2%)	6 (23.1%)	0.18
CKD	45 (8.4%)	30 (4.6%)	4 (2.5%)	13 (6.6%)	0.009	35 (9.8%)	22 (8.5%)	4 (8.2%)	4 (15.4%)	0.68
Hypertension	136 (25.3%)	186 (28.3%)	31 (19.3%)	45 (22.7%)	0.08	155 (43.5%)	118 (45.4%)	16 (12.2%)	6 (23.1%)	0.07
COPD/Asthma	24 (4.5%)	53 (8.1%)	10 (6.2%)	9 (4.5%)	0.05	42 (11.8%)	48 (18.5%)	4 (6.1%)	3 (11.5%)	0.06
**Steroid**	116 (21.6%)	183 (27.9%)	32 (19.9%)	48 (24.2%)	0.04	43 (12.1%)	50 (19.2%)	11 (12.2%)	6 (23.1%)	0.03
**ICU admission**	10 (1.9%)	24 (3.7%)	5 (3.1%)	20 (10.1%)	<0.0001	4 (1.1%)	5 (1.9%)	2 (4.1%)	2 (7.7%)	0.07
**In-hospital death, n (%)**	149 (27.7%)	90 (13.7%)	18 (11.2%)	24 (12.1%)	<0.0001	na	na	na	na	na

P values were obtained by Mann–Whitney U test and ANOVA analysis.

## Discussion

This study investigated the incidence of new-onset diabetes associated with acute SARS-CoV-2 and afterwards, compared to influenza in the Montefiore Health System in the Bronx. Our study sample is diverse and includes many with lower social economic status. The major findings are: i) new-onset diabetes is common at follow-up among COVID-19 patients rendering it a major post-acute sequalae of COVID-19 (PASC); ii) COVID-19 patients are 3.96 times more likely to develop new-onset diabetes compared to influenza during hospitalization but only 1.24 times at follow-up; iii) P-DM is more common among older adults, males, hospitalized patients, patients with preexisting co-morbidities, and those with abnormal admission laboratory tests reflecting increased disease severity and an inflammatory response; iv) predictive models identify risk factors of P-DM in COVID-19 patients with 71.42 ± 2.98% AUC, 78.28 ± 1.72% accuracy; and v) I-DM significantly decreased after COVID-19 vaccine rollout. Although the incidence of new-onset DM across the pandemic could be affected by differences in vaccination rate, strains, COVID-19 testing rate, and population profile, among others, increased risk of diabetes associated with COVID-19 is mediated through disease severity, which plays a dominant role in the development of this post-acute infection sequela.

While COVID-19 causes more severe disease and is affecting older individuals, our findings suggest that the higher rates of P-DM following SARS-CoV-2 compared to influenza (16.7% vs 12.0%) are related to greater clinical severity of the acute infection rather than virus types. The rates of P-DM were similar or even greater after influenza among those with I-DM (37% vs 46.2%) and did not differ significantly from COVID-19 after adjusting for age, sex and markers of severity. Furthermore, the rates of P-DM decreased as severity decreased with each subsequent wave of COVID-19. The decline in severity with each wave may be multifactorial since not only did the virus change but new treatments and vaccines became available, and there may have been immunologic memory even in non-vaccinated patients from prior exposures. Other comorbidities that were more common in patients with COVID-19 such as obesity, pulmonary, cardiac, and renal disease, may have affected disease severity and contributed to the rates of P-DM.

The interrupted time series analysis suggests that the introduction of COVID-19 vaccines, which are associated with a decrease in COVID-19 disease severity, may have played a role in the reduced incidence of I-DM and P-DM although other factors including natural immunity from prior SARS-CoV-2 exposure and differences in viral variants could also contribute to the decreased incidence. Indeed, when grouping data by waves/variants, we observed decreases in the incidence of I-DM and P-DM across the four waves, but no differences in age, sex, and major comorbidities. This could reflect differences in strain virulence, although population profile, COVID-19 testing rates, improvement in available treatments, and as noted above natural immunity or introduction of vaccines in the community could have also contributed.

Incidence of P-DM differed from that of national averages under non-covid pandemic conditions because our cohort excluded patients with pre-DM or those with a prior diagnosis of DM. P-DM in our study may have been impacted by the association with COVID-19 hospitalization and other major comorbidities as well as the demographics of our cohort, which included a large proportion of Blacks and Hispanics, including those who were underserved, who might be at higher risk.

Glucocorticoids were used in patients with severe COVID-19 and those with diabetes and influenza most likely reflecting the high rates of underlying COPD in the patients with influenza. In both viral illnesses their use was strongly associated with I-DM, consistent with the effects of steroids on impairing insulin sensitivity and enhancing hepatic gluconeogenesis. In addition, inflammatory cytokines, which are frequently elevated in the serum of patients with COVID-19, can impair insulin sensitivity and insulin secretion.^25^ Persistent effects of the host response to COVID-19 on adipocytes including the production of adipokines or other inflammatory mediators may account for persistent of DM in some patients as well as the delayed development of P-DM following hospitalization. In addition, beta cell failure or direct damage to hepatocytes may have occurred because of the toxic effects of inflammatory cytokines.^26^ Measurements of these cytokines and adipokines were not available to directly test this hypothesis.

Our findings differ from a previous report that suggested a direct relationship between COVID-19 infection and persistent, post-acute diabetes.^9^ This previous study was limited to Veterans who were predominantly male, whereas we found sex to be a contributing factor in P-DM. Importantly, this previous study did not control for factors such as the severity of the viral illness that seems to be the most significant determinant of post-hospitalization diabetes. To date, most other studies reporting new-onset P-DM were of perspective, case or case serious studies, or cohort studies with no comparison with appropriate controls, making direct comparison challenging. A novelty of our study is that we quantitatively compared new-onset P-DM of SARS-CoV-2 infection with that of influenza, a similar respiratory virus, in the same catchment area. We used predictive model to identify risk factors for developing new-onset P-DM.

There have been several reports suggesting that the SARS-CoV-2 might directly cause diabetes, and some have suggested that the virus might destroy insulin producing β cells directly or indirectly by infecting adipose cells, which produce inflammatory adipokines and enhance insulin resistance.^26^ Clinical experience has been consistent with this notion as large doses of insulin are frequently needed to manage patients in the hospital with diabetes. Based on this concept, it would be expected that diabetes would remit when the acute respiratory illness has resolved, even among those in whom diabetes was discovered in the hospital. Indeed, although the rate of P-DM is higher after COVID-19 than influenza, so was the severity of disease, and most who developed I-DM did not have P-DM at follow-up. This suggests that β cells had not been destroyed but that transient mechanisms associated with inflammatory responses lead to I-DM can resolve in the majority after acute SARS-CoV-2 infection.

### Limitations

There are several limitations in our analysis. Patients who did not return to our health system could not be studied. While it is possible that returned patients were more likely to have more severe COVID-19, our patient data obtained via EMR included those who returned for any medical reasons, including but not limited to regular checkups. Some patients with undiagnosed diabetes or pre-diabetes which could result in some patients being misclassified.

The high percentage of hospitalization is because many COVID-19 patients who came to the emergency department with COVID-19 were hospitalized, especially in the early pandemic. The non-hospitalized patients (enrolled as they presented to our health system and clinics for any medical reason, including regular checkup) were lower than expected because patients without A1c and multiple glucose measures, which were required to confirm DM status, were excluded. This could result in exclusion of some healthier patients and our cohort might not be representative the general COVID-19 patients. We could not distinguish patients hospitalized for COVID-19 from patients who were hospitalized for other indications with incidental COVID-19 because reasons for hospital admission were entered as free text clinical notes and could not be extracted automatically, although incidental COVID-19 was likely low, especially early in the pandemic.

Influenza patients were used as controls as opposed to COVID-19 negative patients to avoid bias by patients who were likely admitted for other serious medical issues (such as trauma, stroke, among others). Influenza testing was not performed on all hospitalized COVID-19 patients, but patients who tested positive for both viruses were rare, consistent with the very low incidence of influenza during the COVID-19 pandemic in 2020 and 2021. This confound is unlikely to alter our overall conclusions.

Incidence of new-onset DM across the pandemic could be affected by vaccination rate, strain, COVID-19 testing rate, population profile, and disease severity, among others. Vaccine status was not reliably recorded if patients received vaccine outside our healthcare system. Vaccines were availability in multiple stages based on age, and multiple doses, types (some requiring one or two shots). Boosters were also administered in the population. Thus, vaccine status is difficult to analyze in detail with respect to outcomes. Although there are published rate of vaccine and waves/strains at the population level in New York City, they are not applicable to our Montefiore cohort which was further filtered by the inclusion and exclusion criteria. Moreover, published data on websites are often bundled together without granular details to allow quantitatively analysis with our data. In addition, COVID-19 testing rate and the profile of the patients across the pandemic could also affect I-DM and P-DM incidence. The effects of these confounds and bias on outcomes are complex, difficult to assess, and not readily discernable from one another.

While our results suggest that hyperinflammation may play a role in new diabetes, data on cytokines and other inflammatory mediators were sparse and not analyzed. We followed patients for ∼3 month after diagnosis on average but recognize that a longer follow-up study is needed. Although our sample sizes were large compared to current published literature, as with any retrospective study, there could be other unintended patient selection bias and unaccounted confounds.

### Conclusions

New-onset diabetes at follow-up (P-DM) was detected in 16.7% of patients who were hospitalized with COVID-19 and who did not have a prior history of pre-diabetes and diabetes. The rates of diabetes were higher in hospitalized compared to non-hospitalized patients with COVID-19. Older patients who were male and with underlying major comorbidities were more likely to have diabetes persist after hospitalization. After adjusting for demographic factors and measures of the severity of illness, the rate of post-infectious diabetes is similar after COVID-19 and influenza. The severity of illness rather than the respiratory virus per se is likely responsible for persistent diabetes. The high incidence of newly diagnosed persistent diabetes during the first wave of the COVID-19 pandemic likely reflects the incidence of severe COVID-19 during this time. This is an exploratory study and prospective studies are needed to confirm these findings. The shear number of COVID-19 patients in the world suggests that new-onset diabetes could be a major public health issue for years to come. Identification of risk factors for P-DM may draw clinical attention for the need for careful follow-up.

## Contributors

J. Wilson – concept and design, collected/verified data, analyzed data, created tables and figures, drafted paper.

J. Lu – concept and design, collected/verified data, analyzed data, created tables and figures, drafted paper.

W. Hou, R. Fleysher - collected/verified data, analyzed data, validated data.

B. Herold, K. Herold – concept and design, edited paper.

T.Q. Duong – concept and design, supervised, edited paper.

## Data sharing statement

Data used is available from the corresponding author upon reasonable request.

## Declaration of interests

Authors declare no conflicts of interest.

## Appendix A Supplementary data

Supplemental Fig. S1

IRB-covid-subprojects
