# Supplementary material for: Incidence of new-onset in-hospital and persistent diabetes in COVID-19 patients: comparison with influenza
Source: eBioMedicine. 2023 Feb 28;90:104487. doi: 10.1016/j.ebiom.2023.104487 (PMC9970376; doi:10.1016/j.ebiom.2023.104487)
Supplement: Supplemental Table S1 [file mmc1.docx]

***Supplemental Table 1.*** *Sensitivity analysis with different levels of prevalence from 15% to 40% positive cases of new-onset P-DM.* As prevalence increased, sensitivity increased but specificity decreased. *PPV: positive predictive values.*

| **COVID-19** |  |  |  |  |  |
| --- | --- | --- | --- | --- | --- |
|  | AUC | Accuracy | Sensitivity | Specificity | PPV |
| 15% | 77.8% [0.65,0.76] | 85.0% [0.83,0.86] | 11.4% [0.08,0.15] | 98.3% [0.98,0.99] | 55.8% [0.44,0.67] |
| 20% | 74.5% [0.72,0.88] | 80.7% [0.77,0.84] | 17.0% [0.11,0.25] | 99.0% [0.97,1.00] | 55.9% [0.47,0.64] |
| 30% | 69.0% [0.64,0.71] | 72.3% [0.70,0.74] | 20.3% [0.16,0.23] | 95.0% [0.94,0.96] | 62.2% [0.55,0.69] |
| 40% | 68.7% [0.62,0.70] | 77.3% [0.74,0.80] | 37.2% [0.29,0.47] | 88.6% [0.85,0.92] | 62.4% [0.57,0.67] |
|  |  |  |  |  |  |
| **Influenza** |  |  |  |  |  |
|  | AUC | Accuracy | Sensitivity | Specificity | PPV |
| 15% | 82.9% [0.70,0.88] | 87.3% [0.83,0.90] | 32.8% [0.21,0.46] | 97.2% [0.95,0.99] | 69.0% [0.49,0.85] |
| 20% | 79.4% [0.66,0.82] | 82.4% [0.78,0.86] | 27.7% [0.18,0.39] | 96.0% [0.88,0.95] | 63.9% [0.52,0.78] |
| 30% | 76.3% [0.65,0.78] | 75.1% [0.70,0.80] | 35.5% [0.27,0.35] | 92.0% [0.88,0.95] | 65.5% [0.52,0.78] |
| 40% | 76.5% [0.64-0.76] | 71.0% [0.65,0.76] | 50.5% [0.41,0.60] | 84.6% [0.78,0.90] | 68.4% [0.57,0.78] |
