# Supplementary figures and images for: Incidence of new-onset in-hospital and persistent diabetes in COVID-19 patients: comparison with influenza
Source: eBioMedicine. 2023 Feb 28;90:104487. doi: 10.1016/j.ebiom.2023.104487 (PMC9970376; doi:10.1016/j.ebiom.2023.104487)

## Slide 1
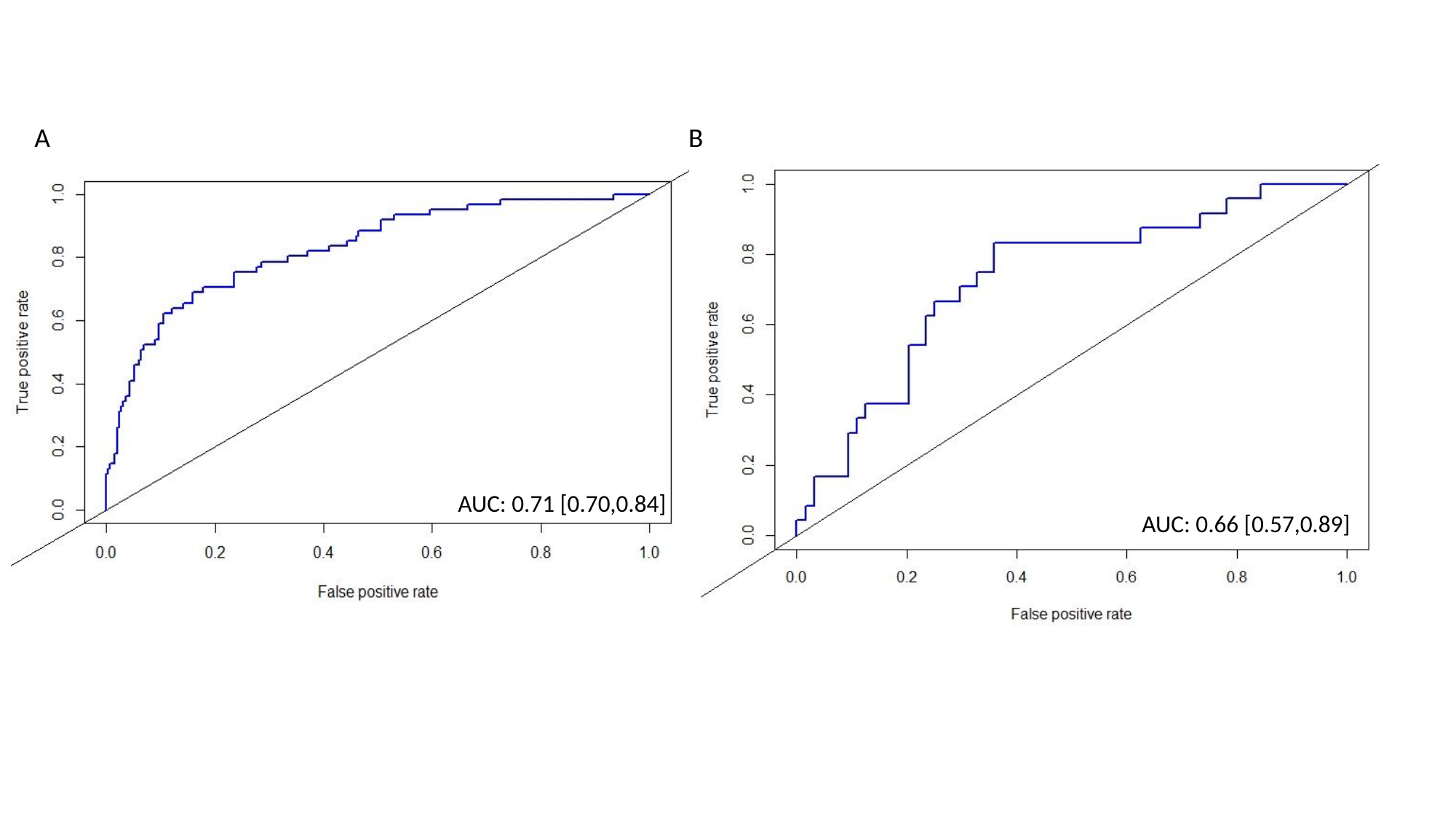

A
B
AUC: 0.71 [0.70,0.84]
AUC: 0.66 [0.57,0.89]

Supplement: Supplemental Fig. S1 [file mmc2.pptx]
