# Supplementary material for: Incidence of new-onset in-hospital and persistent diabetes in COVID-19 patients: comparison with influenza
Source: eBioMedicine. 2023 Feb 28;90:104487. doi: 10.1016/j.ebiom.2023.104487 (PMC9970376; doi:10.1016/j.ebiom.2023.104487)
Supplement: IRB-covid-subprojects [file mmc3.docx]

**IRB amendment to parent IRB for subprojects**

**Subproject title:** Diabetes, hypertension and pulmonary disorders post COVID-19 sequela

**Objective:** This study aims to determine: a) whether COVID-19 related new-onset or worsening of type-2 diabetes occur more frequently among patients with COVID-19 compared to influenza and COVID negative patients, b) whether COVID-19 related new-onset or worsening of hypertension occur more frequently among patients with COVID-19 compared to influenza and COVID negative patients, and c) whether COVID-19 related new-onset or worsening of pulmonary disorders occur more frequently among patients with COVID-19 compared to influenza and COVID negative patients.

**Study period:**  March 11, 2020 to Feb 20, 2022 (may extend as data became available).

For controls, five years of pre-pandemic data are included for comparison (2014-2019, inclusive)

**Exposure**: COVID-19 patients will be identified by polymerase-chain-reaction (PCR) test.

COVID-19 negative patients (controls) will be those tested negative or without a positive PCR test.

**Data sources:** Montefiore Health system EMR via ATLAS/OMOP as described in parent IRB

**Definition of diabetes:** Using pre-COVID-19 pandemic data, we exclude patients with type-2 diabetes or prediabetes ICD10 diagnosis codes, on diabetes medications regardless of diabetes or prediabetes diagnosis, with A1c of 5.7-6.5% (pre-DM) or ≥6.5% (DM) prior to admission, two fasting glucoses of 100-125 mg/dl (pre-DM), a random glucose of 140-199 mg/dl (pre-DM), two fasting glucose readings >126 (DM) or two random glucoses of ≥200 mg/dl prior to admission (DM).

**Definition of hypertension:** The same definitions was used to define hypertension pre-, during COVID-19 hospitalization and post-COVID-19, namely, at least one of the 3 criteria (BP above either 140mmHg systolic or 90mmHg diastolic, on antihypertensive medication at test date, or had an ICD-10 hypertension code). With respect to the BP measurements, average BP 2 weeks before positive test was applied for pre-COVID-19, an average of at least 3 BP measurements for during COVID-19 hospitalization, and the lowest BP post-COVID-19. Patients with PMH of hypertension were excluded.

**Definition of pulmonary disorders:** Newly diagnosed pulmonary disorders were identified based on ICD-10 codes for pulmonary fibrosis, chronic bronchitis, pulmonary embolus, emphysema, COPD, pneumonia, pulmonary edema, pneumothorax and bronchiolitis.

**Major variables to be extracted:** Demographic data included age, sex, race, and ethnicity. Preexisting comorbidities include body mass index (BMI), congestive heart failure (CHF), chronic kidney disease (CKD), hypertension, chronic obstructive pulmonary disease (COPD) and asthma that were designated by ICD10 codes at admission or prior. Steroid treatment, hospitalization status, intensive-care-unit (ICU) admission, and mortality will also be extracted. Admission vital signs and laboratory data collected from hospitalized patients include temperature, systolic blood pressure (SBP), oxygen saturation (SPO_2_), lactate dehydrogenase (LDH), brain natriuretic peptide (BNP), creatinine (Cr), C-reactive protein (CRP), ferritin (FERR), D-dimer (DDIM), troponin-T (TNT), alanine aminotransferase (ALT), white-blood-cell count (WBC), lymphocyte count (Lymph), and prothrombin time (PT). Additional variables (including imaging data) may be added if appropriate.

**Outcomes:** Primary outcomes will be the incidence of new-onset type-2 diabetes and hypertension while in the hospital, and the incident of new-onset persistent diabetes and hypertension at ~3-month (and beyond) for patients who returned to the hospital system. Primary outcomes will include the incidence of newly diagnosed pulmonary disorders

**Statistical analysis:**  Categorical variables will be compared using χ^2^ or Fisher’s exact tests, and continuous variables will be compared using t-test or Mann-Whitney U test whenever appropriate. We will use logistic regression to predict P-DM with demographics, comorbidities, and lab values. Univariate analysis will be performed first, and then top significant variables will be selected to be entered into the multivariable model. Odds ratios and 95% confidence intervals will be calculated based on the logistic regression. Incidents of I-DM and P-DM will also be compared over time during Covid-19 pandemic. P value less than 0.05 will be considered as statistical significance. All statistical analysis will be performed using SAS (Version 9.4, Cary, NC, USA).

**Predictive model:** Logistic regression or machine learning will be used to build the predictive model. Sample size was based on availability of subjects. Univariable analysis was performed using each variable separately (demographics, comorbidities, and lab values, Imputation will be done for data missing <15%. The top laboratory variables will first be identified based on P-values. These top laboratory variables will then be combined with demographics, comorbidities to the logistic model to predict outcomes. This approach is adopted to avoid overfitting. Model performance was evaluated using area under the receiver operating characteristic curve with five-fold cross validation.
